# A preliminary study of further attempt at the development, testing and application of an independent primary screening stool card

**DOI:** 10.1038/s41598-022-26649-2

**Published:** 2022-12-21

**Authors:** Huimin Cai, Hongliang Chen, Yang Gao, Qianqian Huang, Chengqian Lv, Xueyu Cang, Jihan Qi, Kunpeng Luo, Shizhu Jin

**Affiliations:** Department of Gastroenterology and Hepatology, The Second Affiliated Hospital of Harbin Medical University, 246 Xuefu Road, Nangang District, Harbin, 150086 China

**Keywords:** Health care, Medical research

## Abstract

Stool characteristics are of great value to assess diseases, but patients knew little. E-learning applied in health popularization and patient education is booming. In China, WeChat applets has advantages of abundant users, convenient access and low cost, which may be a great media in patient education on stool. This preliminary study aims to develop and evaluate a stool card WeChat applet. We collected stools images during 2020 to 2022 in the Department of Gastroenterology and Hepatology in the Second Affiliated Hospital of Harbin Medical University, constructed a stool card applet named the Doctor Friend Primary Screening Stool Card (DFPSSC) and evaluated it. Eligible participants were divided into the applet, traditional paper media and control group. We implement a series of tests to evaluate the effectiveness. 20 clinicians and participants using the DFPSSC completed a questionnaire to evaluate the usability. We developed the DFPSSC for an E-learning approach. Of 108 volunteers, 97 completed the DFPSSC learning. No significant pretest differences were found among the three groups (*P* = 0.303). Applet group had significantly higher posttest scores than pretest scores in intervention (*P* < 0.001, d = 1.68) and simulation (*P* = 0.006) test, and it had higher scores than other two group (*P* < 0.001). 63% participants and 59% clinicians strongly agree or agree to the usability of DFSSC. This preliminary study verified that the DFPSSC can effectively improve participants’ knowledge of feces, making it an effective clinical tool for patient education and the avoidance of treatment delay.

## Introduction

The characteristics of feces are helpful for gastroenterologists to diagnose and evaluate the severity of disease in clinical practice. Stool card is the significant media for popularization of science of feces among patients. In 1994, Japan used stool color card (SCC) to screen for congenital biliary atresia (BA)^1^. In 2004, Taiwan Province of China introduced the concept of SCC and fully promoted application^2^. Studies showed that Taiwanese SCC shortened the treatment time for BA, increase the implementation rate of the Kasai procedure within 60 days after birth, reduce the hospitalization rate and mortality of BA^3^, and improve the 5-year prognosis of BA^4^. Similarly, a 19-year cohort study from Japan found that stool color cards could improve the timing of Kasai procedure and the long-term survival rate of primary liver^5^. Since then, the reliability and repeatability of SCC have been further verified in other regions^6–9^. From another perspective, stool color card is a low-cost and cost-effective screening tool, which has been confirmed by Douglas Mogul et al.^10^. These benefits are realized by improving parents' awareness of infant feces through stool color cards^11^. And lack of correct awareness of feces may lead to adverse consequences, Laine L’s study showed melena without hematemesis was the most important independent factor leading to patients' delay in seeking treatment^12^. Therefore, it’s beneficial to emphasize the importance of feces to both clinician and patients, which enhanced the accuracy of therapy and shortened the disease progression. However, the limited amount of information contained in the card and the trend of sinking paper media prevents its further popularization.

Nowadays, patient education has become an important part of clinical work for the sake of patient compliance and better clinical results^13,14^. E-learning, defined as any type of educational media provided in electronic form, has gradually become the principal approach to explore new educational methods^15^. E-learning has many advantages, such as lower communication costs, richer information displays and more convenient access compared with traditional education methods^16,17^. Mobile health projects used for patient education and the professional improvement medical staff^18–20^ have applied in many chronic diseases and bad habits like irritable bowel syndrome^21^, diabetes^22,23^, smoking^24^, and overweight and obesity^25^. Japan published an app in 2017 that can evaluate whether newborns have abnormal fecal color to screen BA^26^, however, function of which is still simple. Therefore, a full-featured and abundant-types of diseases stool card APP is necessary.

WeChat owned exceeded 1.1 billion users, which is most commonly used social media in China. The WeChat applet is a program running on the WeChat platform. Compared with traditional mobile phone applications, the WeChat applet has the advantages of not required to download for use, having less traffic consumption and having low promotion cost, which played an important role in China's epidemic prevention and control during the COVID-19 pandemic.

Therefore, we will develop a new stool card. The difference between it and Taiwan fecal color card is that it has more classification dimensions, can be applied to more diseases and older age groups, contains more information, and is presented in the form of applet. It would allow people with little fecal knowledge, including healthy people and patients to compare their feces with the picture on the applet to interpret the information contained in their feces. This study aimed to (1) develop a stool card WeChat applet, (2) evaluate the effectiveness and usability of the stool card applet.

## Method

### Development and introduction of a stool card applet

We collected fecal images in the relevant departments of the Second Affiliated Hospital of Harbin Medical University. We used most of the pictures to develop the applet and retained some for subsequent testing. Then, the classification logic was established according to the color and shape of feces. We divided the color of feces into yellow, brown, green, white, red and black. The shape of feces was divided into 7 types according to the Bristol Stool Form Scale (BSFS)^27^.

We classified the collected feces according to the classification logic and analyzed each kind of feces to inform patients about their feces normality or abnormality, the diseases they may be indicative of, and the appropriate next steps. The information provided was reviewed by senior and experienced physicians who have been working in the Second Affiliated Hospital of Harbin Medical University for many years to ensure the accuracy of the information provided.

**Figure 1:**
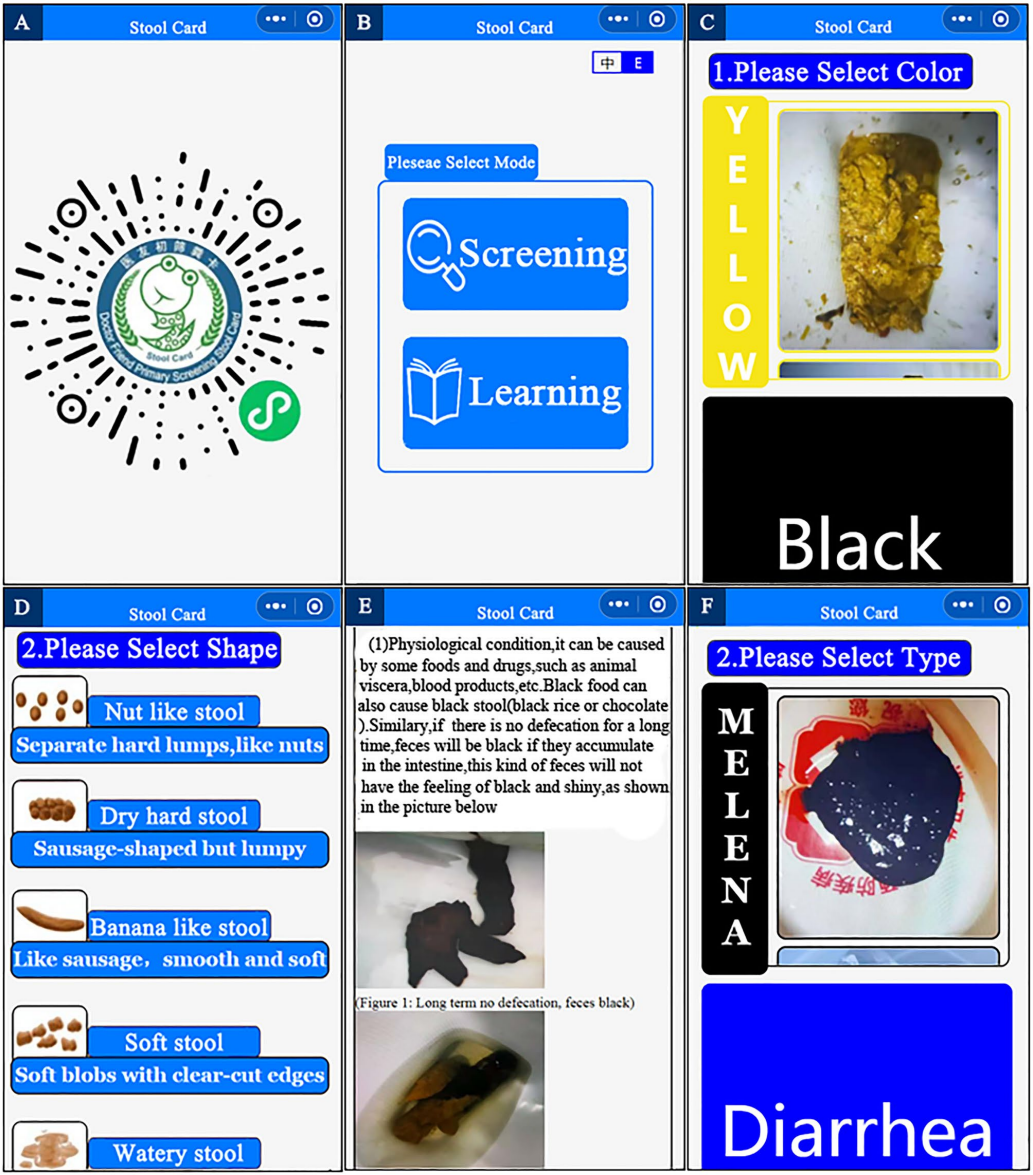
Interface display of Doctor Friend Primary Screening Stool Card. (**A**) QR code display of applet. (**B**) Mode selection interface. (**C**) Screening mode—color selection interface. (**D**) Screening mode—shape selection interface. (**E**) Fecal information display interface. (**F**) Learning mode interface.

Then, WXML language and JavaScript was used to develop the WeChat applet, which is named the Doctor Friend Primary Screening Stool Card (DFPSSC). The applet supports both Chinese and English. The interface of the WeChat applet is presented in Fig. 1.

### Study 1: preliminary validation of effectiveness of DFPSSC

#### Participant recruitment and grouping method

We recruited freshmen from Harbin Medical University for testing. Freshman at this university have not systematically learned relevant professional knowledge, and their cognition of feces is therefore at the same level as that of nonmedical professionals. We randomly selected a dormitory building for freshmen and selected 6 bedrooms on different floors (a total of 3 floors, 18 bedrooms and 108 people).

People who agreed to participate were tested, excluding those who scored higher (score greater than 80%), because this project was aimed at those who did not have ample knowledge about feces. The remaining people were divided into three groups according to their respective floors. The first group was the group using the stool card applet.

#### The detail of the test

All subjects meeting the inclusion criteria will be tested again after the trial intervention. The tests focused on the identification of common stool types, diseases indicated by feces and countermeasures in different situations. The tests consist of 30 multiple-choice questions, each of which had 4 response options with single or multiple correct answers. Each question had a value of two points, for a total score of 60 points.To ensure that the difficulty of the pretest was consistent with the post test, questions and the question options were the same, but the question order was changed. The participants were not informed of the answer after each test. Finally, four clinicians from the Department of Gastroenterology of the Second Affiliated Hospital of HMU reviewed the test questions to ensure that they were suitable for this experiment. The questions list is provided as Multimedia Appendix 1.

Although the subjective factors of raters were excluded when testing in the form of multiple-choice questions, participants might have chosen the correct answer without mastering the related knowledge because of their skills and the hints provided in the options in multiple-choice questions. Therefore, we simulated simple situations in which abnormal stool occurred and then asked participants to give solutions to these situations. Finally, their answers were scored by a clinician. The stimulation test is provided as Multimedia Appendix 2.

The test was conducted in the dormitory of the subject to ensure fairness, each test was carried out under our supervision. Participants were required to keep a certain distance from each other, and they were not allowed to read any materials or use electronic equipment.

#### Implement method

We posted the QR code for the stool card applet on the door of the public toilet on the floor so that the participants in this group could easily use the applet when defecating. The second group was the traditional paper group. A paper stool card was distributed to each selected bedroom; the content of the paper version was the same as that of the applet. The third group was the control group without any intervention.

One month after the pretest, the participants were tested again, and the results of the post test were compared with those of the pretest. The comparison of the two tests and the simulation test results will jointly verify the effectiveness of DFPSSC.

### Study 2: preliminary validation of usability of DFPSSC

#### Participants selection and implement method

After completing the effectiveness test, the subject group members using the applet will complete a questionnaire to assess the usability of the applet. Then, we also invited 20 clinicians with rich clinical experience. They were all engaged in the diagnosis and treatment of gastrointestinal diseases, and all had more than 5 years of work experience. After using the applet for 1 week, they will finish a questionnaire designed for them.

#### The detail of questionnaire

On the basis of previous practice, Andrea Fairman et al. designed MAUQ, which categorizes the types of app by two dimensions^28^. The first dimension is interaction modes (interactive or standalone), and the second dimension is target users of app (patients or health services providers), these two dimensions divide the app into 4 kinds, then MAUQ provides 4 questionnaires suitable for different situations based on different classifications. According to the above classification, the stool card applet adopted the questionnaire designed for standalone mHealth apps (the Cronbach alpha = 0.914). There are 17 items in the questionnaire, covering three aspects (Ease of Use, Interface and Satisfaction, Usefulness). Patients and clinicians took the different version of questionnaire to carry out the research. Their differences are reflected in the items in Usefulness. The version for patients focused on whether users can get better health services, and the one for clinicians focused on whether users can practice better health services. And the items of the questionnaires were rated on a Likert scale (1–5 points: strongly disagree to strongly agree).

### Statistical analysis

Continuous variables are expressed as the means with standard deviation (SD). Categorical variables are reported using frequencies and percentages. The normality assumption for test scores was verified with the Shapiro–Wilk test. When the data approximately conformed to a normal distribution, if they satisfied the assumption of homoscedasticity, one-way analysis of variance (ANOVA) was performed to determine the significance of differences, and Tukey’s test was used for post hoc testing; if the assumption of homoscedasticity was not met, Welch's ANOVA was performed to determine the significance of the differences, and the Games-Howell test was used for post hoc testing. Non normally distributed data were tested with Kruskal–Wallis ANOVA to determine the significance of differences in distribution and Bonferroni’s test adjusted for *P* values. The t test for paired samples was performed to compare the efficiency of the intervention. *P* values < 0.05 were considered significant. All data were analyzed using Statistical Package for Social Sciences 26.0 (SPSS, Inc. Chicago, Illinois, USA).

### Ethical approval

Our study has been approved by the Ethics Committee of the Second Affiliated Hospital of Harbin Medical University (KY2020-270). All procedures performed in studies involving human participants were in accordance with Helsinki declaration.

### Informed consent

Informed consent was obtained from all individual participants included in the study.

## Results

### Study population

**Figure 2:**
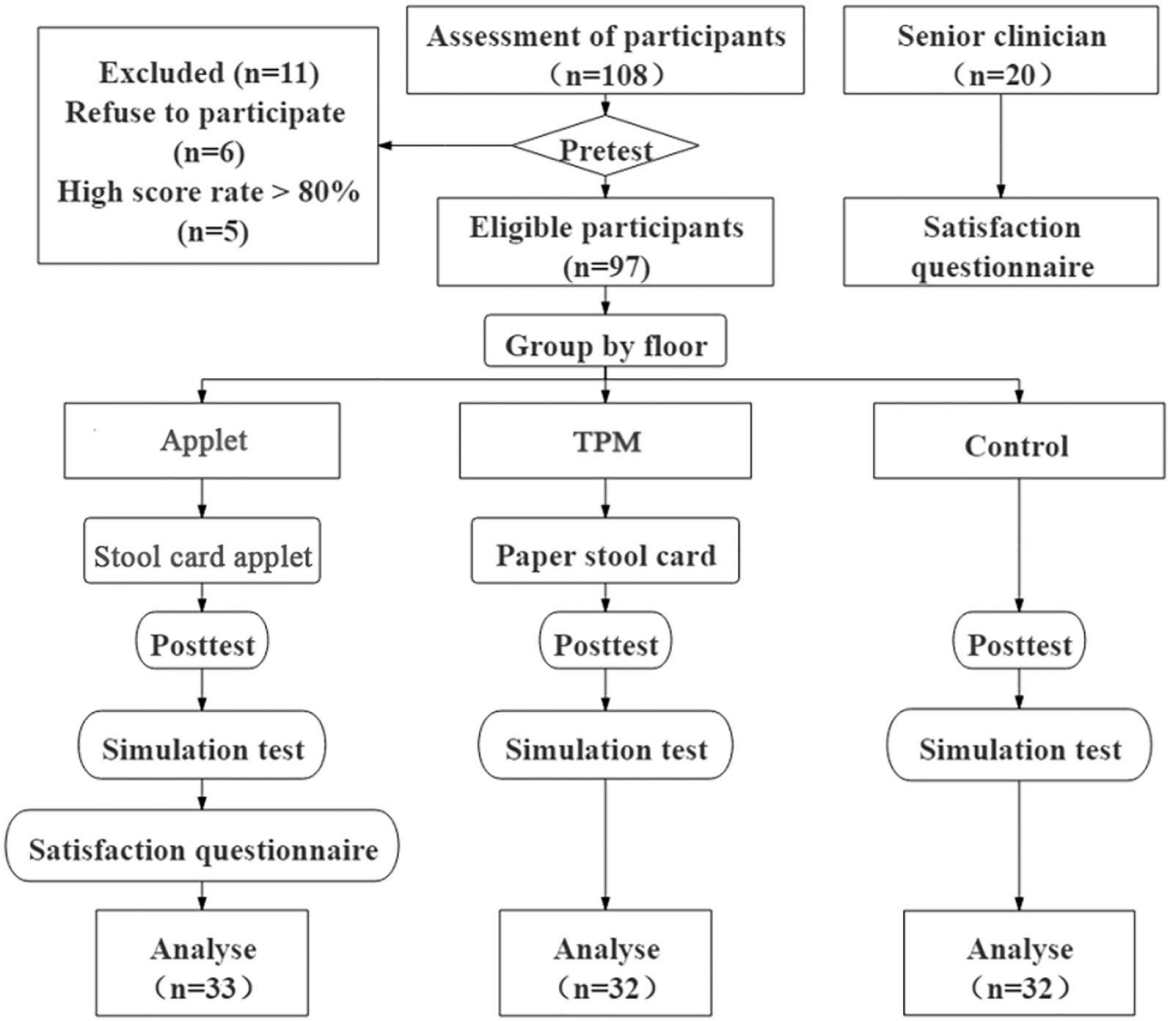
Flowchart showing details of participant selection, grouping, and testing. Applet: applet group; TPM: traditional paper media group.

Among 108 freshmen majoring in clinical medicine from Harbin Medical University, 11 students were excluded from the study, including 6 who declined to participate and 5 who scored at least 80% on the pretest. According to the floors of the dormitory, participants were grouped into the applet group (n = 33), traditional paper media group (n = 32) and control group (n = 32). It should be noted that in the dormitories of Harbin Medical University, each floor shared a public toilet, which facilitated our research, but also led to the fact that the student samples we selected were all male.We also invited 20 physicians who have been working in the Department of Gastroenterology of the Second Affiliated Hospital of Harbin Medical University for many years The detail is shown in Fig. 2. The characteristic of participants is shown in Table 1.

**Table 1:** Demographic and characteristics of participants.

Characteristics	Students (n = 97)				Physicians (n = 20)
	Applet (n = 33)	TPM (n = 32)	Control (n = 32)	P value	
Age (mean, range)	17.97 (17–19)	17.88 (17–18)	18.06 (18–20)	0.086	30–55 (37.95)
Gender (male/female)	33/0	32/0	32/0	–	9/11
**Scores (mean, range)**					
Pretest score	33.39 (26–42)	33.88 (22–44)	34.97 (26–46)	0.436	
Posttest score	40.67 (28–58)	40.44 (30–56)	34.13 (22–42)	< 0.001	
Test score	45.97 (25–62)	38.16 (24–64)	30.38 (20–48)	< 0.001	
**Academic record (n, %)**				0.969	
Grade A	15 (45)	14 (44)	15 (47)		
Grade B	18 (55)	18 (56)	17 (53)		
**Professional (n, %)**					
Attending physician					12 (60)
Deputy chief physician					4 (20)
Chief physician					4 (20)
**Seniority (n, %)**					
5–10 years					8 (40)
10–15 years					8 (40)
> 15 years					4 (20)

TPM: Traditional paper media; Grade A presents ranking in the top 50% of the grade; Grade B presents ranking at the bottom 50% of the grade.

### Results of study 1: validation of effectiveness of DFPSSC

#### Comparison of pretest and posttest scores among the app group, paper group and control group

**Figure 3:**
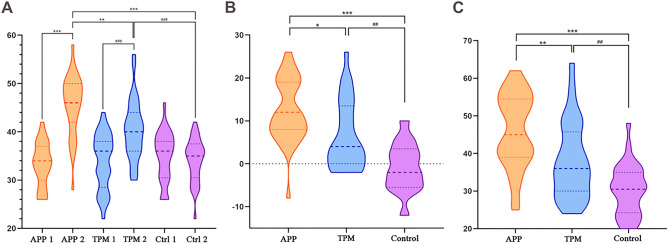
Violin plots. (**A**) The compare of pre- and post-test scores of three groups. (**B**) The progress or retrogress (post-test score – pre-test score) of three groups. (**C**) The compare of scores of simulation test of three groups. (*P < 0.05, **P < 0.01, ***P < 0.001). TPM: traditional paper media group.

Pretest scores and post test scores among the three groups conformed to an approximate normal distribution (P > 0.05). There were no significant differences in pretest scores among the three groups (F = 1.211, P = 0.303). In the control group, pretest and posttest scores were not significantly different (P = 0.392). After intervention, the applet group (mean ± SD: 45.67 ± 6.24 vs. 33.39 ± 4.46, P < 0.001, d = 1.68) and traditional paper group (40.44 ± 6.20 vs. 33.88 ± 5.701, P < 0.001, d = 0.83) had significantly higher posttest scores than pretest scores. The posttest scores among the three groups were significantly different (F [2, 61.115] = 38.285, P < 0.001). The interventions using the applet (45.67 ± 6.24 vs. 34.13 ± 4.46, P < 0.001) and the traditional paper format (40.44 ± 6.20 vs. 34.13 ± 4.46, P < 0.001) were effective. In addition, the applet group showed a more noteworthy improvement in their scores than the traditional paper group (P = 0.001) (Fig. 3A).

The difference values (post test score – pretest score) in the applet group (mean ± SD: 12.53 ± 1.29), traditional paper group (6.56 ± 1.39) and control group (0.84 ± 0.97) were significantly different between the pairs of groups (PApp-Pape r = 0.013, PApp-Control < 0.001, PPaper-Control < 0.001, P < 0.001) (Fig. 3B).

#### Validating the efficiency of the intervention by simulation test

The results were the same as those of the posttest. The applet group (mean ± SD: 45.97 ± 9.81 vs. 30.38 ± 6.64, P = 0.006) and traditional paper group (38.16 ± 10.15 vs. 30.38 ± 6.64, P < 0.001) had a stronger capability to analyze stools than the control group (F [2,94] = 24.31, P < 0.001, η^2^ = 0.341, ω^2^ = 0.32). The scores were significant difference in each two groups compared by Games-Howell.

In addition, the applet group had a stronger capability than the traditional paper group (P = 0.001) (Fig. 3C).

The description of statistical analysis is shown in Supplementary Materials 1.

### Result of study 2: validation of usability of DFPSSC

**Figure 4:**
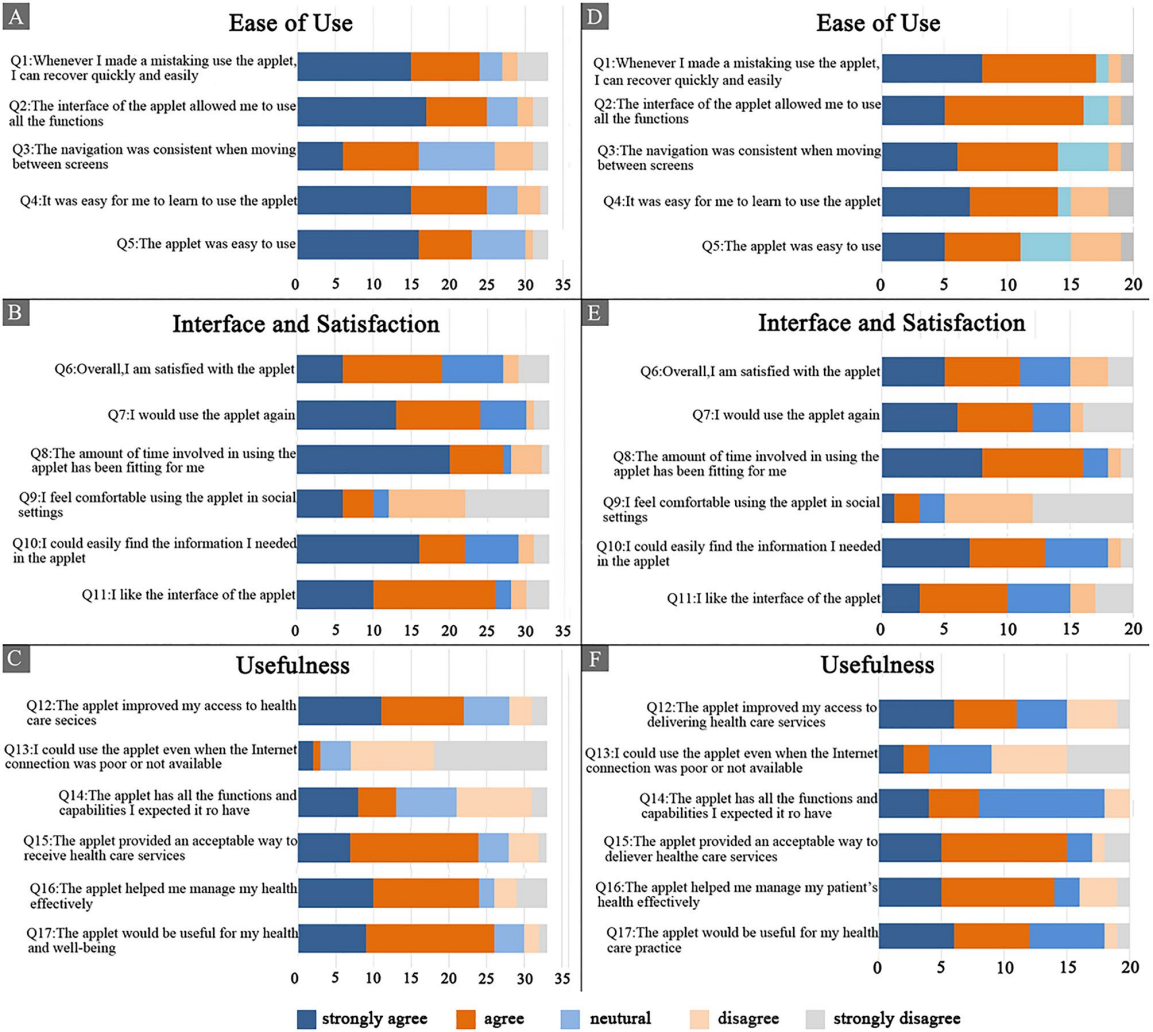
The results of the satisfaction questionnaire. (**A**) The questions of ease of use designed for patients. (**B**) The questions of interface and satisfaction designed for patients. (**C**) The questions of usefulness designed for patients. (**D**) The questions of ease of use designed for health services providers. (**E**) The questions of interface and satisfaction designed for health services providers. (**F**) The questions of usefulness designed for health services providers.

The results are shown in Fig. 4. The kinds of color represent different attitudes, and the length of the rectangle represents the number of people.

In the questionnaire designed for standalone mHealth apps (patient version), the average proportion of participants who strongly agreed or agreed with the topic of the ease of use of the applet, namely, Q1–Q5, was *68%*, with the highest proportion of participants agreeing with Q2 (The interface of the DFPSSC allowed me to use all the functions) and Q4 (It was easy for me to learn to use the DFPSSC), both at 75%, and the lowest proportion agreeing with Q3 (The navigation was consistent when moving between screens) at 49%. For Q6 to Q11, that is, the topic of interface and satisfaction, the mean proportion of participants who strongly agreed or agreed with the items was *64%*, and the highest proportion was 81%, for Q8 (The amount of time involved in using the DFPSSC was suitable for me). Q9 (I feel comfortable using the DFPSSC in social settings) had the lowest proportion (30%). For the topic of the usefulness of the applet (Q12–Q17), the average proportion of participants who strongly agreed or agreed was *56%*. The highest proportion and the lowest proportion were for Q17 (The DFPSSC would be useful for my health and well-being) (79%) and Q13 (I could use the DFPSSC even when the internet connection was poor or not available) (9%), respectively.

Correspondingly, in the questionnaires designed for standalone mHealth apps (health service provider version), for the ease of use topic (Q1-Q5), the highest proportion and the lowest proportion of strongly agree or agree responses were for Q1 (Whenever I made a mistake using the DFPSSC, I could recover quickly and easily) (85%) and Q5 (The DFPSSC was easy to use) (55%), respectively, with a mean of *72%*. Regarding the interface and satisfaction topic (Q6-Q11), the mean proportion of strongly agree or agree responses was *54%*, while the highest proportion was for Q8 (The amount of time involved in using the DFPSSC was suitable for me) (80%), and the lowest was for Q9 (I feel comfortable using the DFPSSC in social settings) (50%). For the topic of usefulness, the average proportion of participants who strongly agreed or agreed with the responses was *53%*, with the highest proportion for Q16 (The DFPSSC helped me manage my patients’ health effectively) (75%), and the lowest was for Q13 (I could use the DFPSSC even when the internet connection was poor or not available) (20%). The results of the satisfaction questionnaire are also detailed in Multimedia Appendix 3.

In addition, among freshmen, we divided them into two groups according to their academic achievements, sorted out the distribution of satisfaction, and measured it with chi-square test. There was no significant difference between the two groups (P = 0.208). In the group of clinicians, we took their professional titles as grouping factors, and found no significant difference (P = 0.281). The description of the chi-square test in shown in the Multimedia Appendix 4.

## Discussion

### Principal findings

The first major outcome of this study was the development of a stool card WeChat applet for all ages and additional diseases. A patient’s higher knowledge of disease is important and meaningful to improve their compliance^29–31^. A lack of patient knowledge may lead to misunderstandings of physicians’ decisions, hindering treatment. Regarding the characteristics of stool, through practice and research^12^, we found that patients have little knowledge of them and even ignore them frequently, so they cannot provide accurate information. For example, certain foods can cause a change in color of stool that, without the knowledge related to it, may cause unnecessary expenditure. Formally, although SCC has also achieved a shift toward digitalization^32^, the cost of promoting a not-for-profit app is high, with little success. The DFPSSC contains more content than an SCC, and WeChat's popularity and the ease of access to the stool card applet drastically reduces its promotion cost. Thanks to the convenience of WeChat applet, we will post the QR code in toilets in public places, such as schools, hospitals, subway stations, etc., so that DFPSSC can be used when defecating Secondly, we will display and spread it through online media (such as Tiktok and Weibo). Any person who owns a smart phone and installs WeChat can use DFPSSC and obtain information related to their own feces.

The second principal result was the preliminary verification of the effectiveness of the DFPSSC and comparison of the differences among the three groups through tests. Before the intervention, we found that there was no obvious distinction in participants’ knowledge regarding feces through the pretest. After a month of independent learning, the participants were tested again, and it was apparent that the test scores of the groups using the stool cards, both the applet group and the traditional paper group, were significantly improved compared with those of the control group, which shows that the stool card was effective in improving users' cognition about feces. In addition, the improvement in the applet group’s score was significantly better than that of the traditional paper medium group. This result shows that the stool card applet was more efficient in improving users' cognition about feces. To further verify the effectiveness of the stool card applet, we adopted simulation tests, and the results also showed that the DFPSSC was effective and better than the traditional paper medium in helping participants perform better in different situations. In short, the DFPSSC provides users with abundant information about feces to help them understand the characteristics of their own feces, which enables them to better understand doctors' clinical decisions. In addition, the DFPSSC can better provide health education for patients without limiting the amount of information presented because of its portability and accessibility.

In the third main result,we get the preliminary evaluation on the usability of DFPSSC. In the version designed for patients, users were more satisfied with the simplicity of using the DFPSSC, and regardless of the version, participants approved of the health benefits of the DFPSSC. However, they were not willing to use the DFPSSC in publicfirst, because it is embarrassing to browse fecal pictures in public and, second, because the DFPSSC required the availability of the internet, and they were not satisfied with the use experience when the network conditions were poor. Finally, participants wished that the DFPSSC could have more functions, such as the intelligent recognition of feces. In the version for the health service providers, we obtained similar results, with the difference being that their overall satisfaction with the function of the DFPSSC was higher. This might be due to the different perspectives due to by their roles.In the freshmen test group, different academic year grades can, to a certain extent, represent the seriousness of students' assigned tasks. Therefore, we conducted chi-square test on students with different academic year grades, and found no significant difference in their evaluation of usability. Similarly, in the group of doctors, we measured by groups according to their different professional titles, and obtained similar results.

## Limitations

Our study also has limitations. DFPSSC is still in the early construction stage, therefore still need wide range of people to verify its universality. And our tests are limited to specific populations and the sample size is small, making our research lack representativeness. Therefore, this study potentially needs a richer population composition and sample size to verify and guarantee its effectiveness and usability. We preliminarily verified its effectiveness and accessibility, provide direction and evidence for our subsequent work in order to update to DFPSSC 2.0.

## Conclusions

The results of this study show that the DFPSSC can effectively improve users' cognition about feces and has higher efficiency than traditional paper media. The DFPSSC can be a meaningful supplement to existing forms of patient education, reduce the communication barriers between patients and doctors, and promote the smooth progress of clinical work. The DFPSSC is easy to use and is good for the user’s health, but its function and accessibility are just acceptable. Therefore, we will continue to upgrade the DFPSSC so that it can have a more satisfactory interface, richer and accurate information and more intelligent usage.

## Supplementary Information

Supplementary Tables.

Supplementary Information 2.

Supplementary Information 3.

Supplementary Information 4.

Supplementary Information 5.

Supplementary Information 6.

Supplementary Information 7.

Supplementary Information 8.

## Data Availability

All data generated or analysed during this study are included in this published article (and its supplementary information files).

## Abbreviations

SCC: Stool color card
BA: Biliary atresia
DFPSSC: Doctor Friend Primary Screening Stool Card
BSFS: Bristol Stool Form Scale
TPM: Traditional paper media
MAUQ: MHealth App Usability Questionnaire
SD: Standard deviation
ANOVA: One-way analysis of variance
