# Supplementary material for: A preliminary study of further attempt at the development, testing and application of an independent primary screening stool card
Source: Sci Rep. 2022 Dec 21;12:22046. doi: 10.1038/s41598-022-26649-2 (PMC9768403; doi:10.1038/s41598-022-26649-2)
Supplement: Supplementary file 1 — Supplementary Tables. [file 41598_2022_26649_MOESM1_ESM.docx]

Supplementary Materials

Appendix. ……

Supplementary Tables

Table S1. Normality test for scores. Abbreviation: TPM, traditional paper media group.

Table S2. Table S2. Details for One-way ANOVA and Kruskal-Wallis test in this study. Abbreviation: TPM, traditional paper media group.

Table S1. Normality test for scores.

| Scores | P value for Shapiro-Wilk test |
| --- | --- |
| Applet |  |
| Pretest | 0.15 |
| Posttest | 0.144 |
| Difference | 0.114 |
| TPM |  |
| Pretest | 0.102 |
| Posttest | 0.364 |
| Difference | 0.005 |
| Control |  |
| Pretest | 0.122 |
| Posttest | 0.165 |
| Difference | 0.397 |
| Test |  |
| Applet | 0.268 |
| TPM | 0.09 |
| Control | 0.372 |

Abbreviation: TPM, traditional paper media group.

Table S2. Details for One-way ANOVA and Kruskal-Wallis test in this study.

|  | Pretest | Posttest | Difference | Test |
| --- | --- | --- | --- | --- |
| Method | One-way ANOVA | One-way ANOVA | Kruskal-Wallis test | One-way ANOVA |
| Test of Homogeneity of Variances |  | 0.26 |  | 0.02 |
| F (df1, df2) |  | 38.285 (2, 61.115) |  | 24.31 (2, 94) |
| P value | 0.449 | 1.66E-11 | 2.10E-09 | 1.55E-09 |
| Multiple Comparison |  | Games-Howell | Bonferroni | Turkey |
| Applet vs. TPM |  | 0.00342 | 0.012517 | 0.00209 |
| Applet vs. Control |  | 5.12E-09 | 8.07E-10 | 6.37E-09 |
| TPM vs. Control |  | 0.000054 | 0.001847 | 0.002386 |

Abbreviation: TPM, traditional paper media group.
