# Supplementary material for: A preliminary study of further attempt at the development, testing and application of an independent primary screening stool card
Source: Sci Rep. 2022 Dec 21;12:22046. doi: 10.1038/s41598-022-26649-2 (PMC9768403; doi:10.1038/s41598-022-26649-2)
Supplement: Supplementary file 2 — Supplementary Information 2. [file 41598_2022_26649_MOESM2_ESM.docx]

Fecal Cognition Assessment Test

Name: Score:

**Single choice questions (2 points for each question, 30 questions in total, 60 points in total)**

1. Which of the following feces is melena (tarry stool)（）？

A.
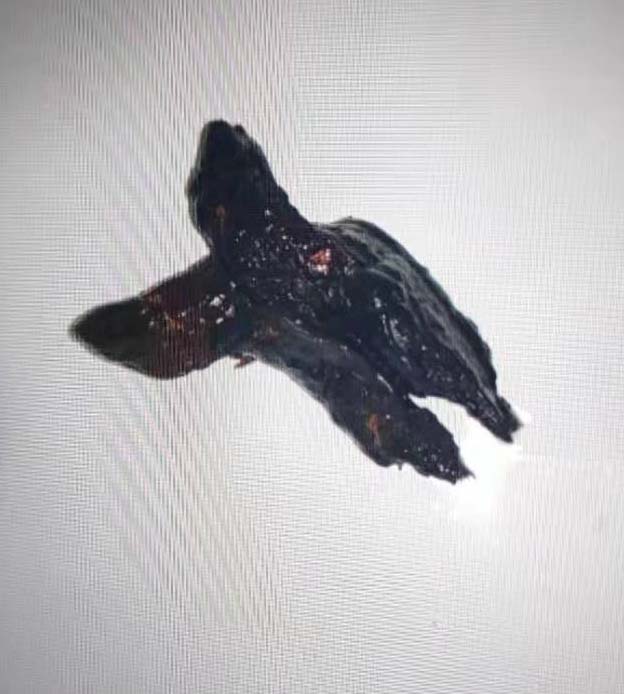


B.
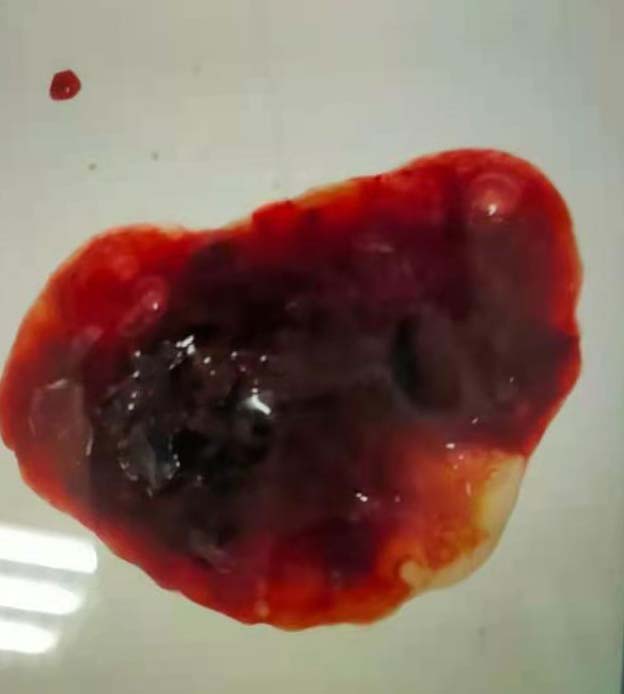


C.
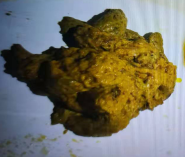


D.
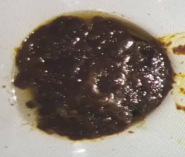


1. Under pathological conditions, what are the reasons for melena?（）
2. Upper gastrointestinal bleeding
3. Lower gastrointestinal bleeding
4. Eating chocolate
5. Gastritis
6. Which of the following foods can cause black stool：（）
7. Duck blood
8. Pork
9. Glutinous rice
10. Chicken gizzard
11. What are the causes of black stool in patients with a history of liver cirrhosis or chronic liver disease（）
12. Esophagogastric variceal hemorrhage
13. Gastric ulcer or duodenal ulcer
14. Acute gastritis
15. Gastric cancer
16. No history of liver, regular history of epigastric pain (such as hunger pain), the cause of black stool may be（）
17. Esophagogastric variceal hemorrhage
18. Gastric ulcer or duodenal ulcer
19. Acute gastritis
20. Gastric cancer
21. What should an elderly people without history of liver disease vigilant in case of black stool（）
22. Esophagogastric variceal hemorrhage
23. Gastric ulcer or duodenal ulcer
24. Acute gastritis
25. Gastric cancer
26. What measures should be taken in time after the occurrence of black stool?（）
27. Temporary fasting and water, while reducing activity
28. Normal eating
29. Neglect of feces
30. Normal activity or even vigorous activity
31. What is the best way to check the cause of black stool?（）
32. Gastroscope
33. Gastrointestinal radiography
34. Abdominal CT
35. Ultrasound
36. Which of the following situations can consider no active bleeding?（）
37. No black stool for 3 days or yellow stool
38. No black stool for 1 day or yellow stool
39. Normal blood pressure and heart rate
40. The patient felt that the bleeding had stopped
41. Which of the following stool shapes is considered to be caused by constipation?（）
42.
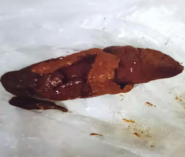

43.
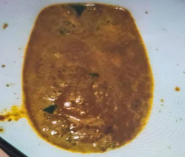

44.
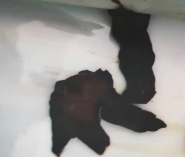

45.
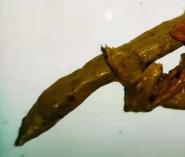

46. Which of the following stool shapes is considered to be caused by diarrhea?（）
47.
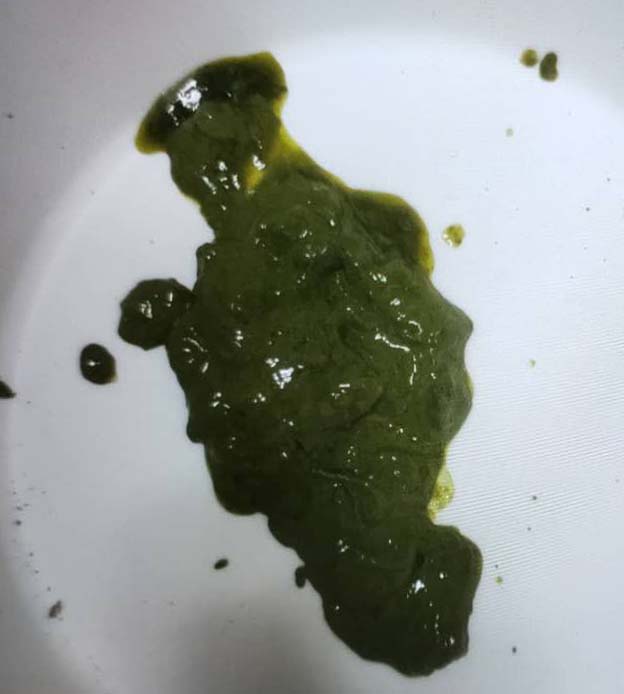

48.
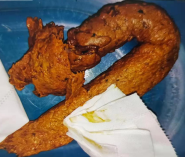

49.
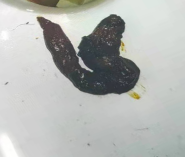

50.
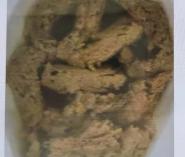

51. Which of the following stool shapes is a normal stool?（）
52.
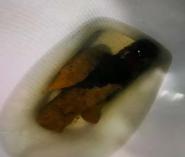

53.
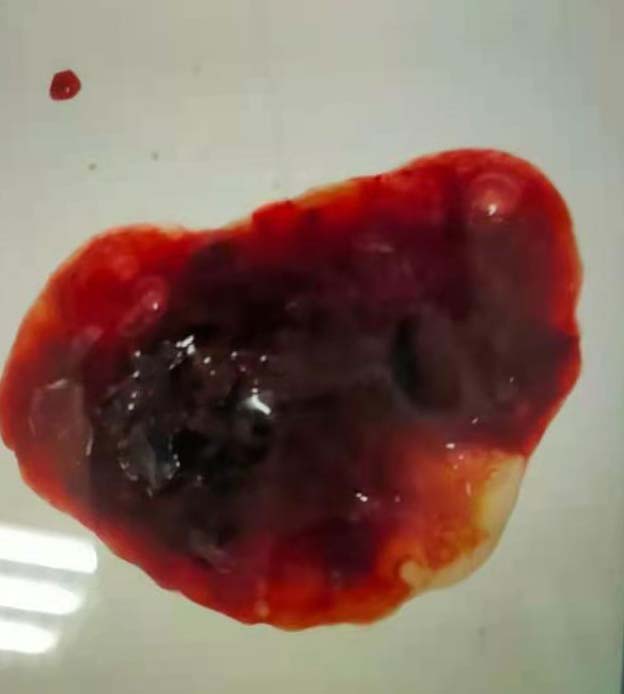

54.
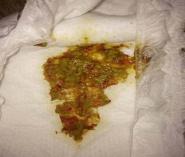

55.
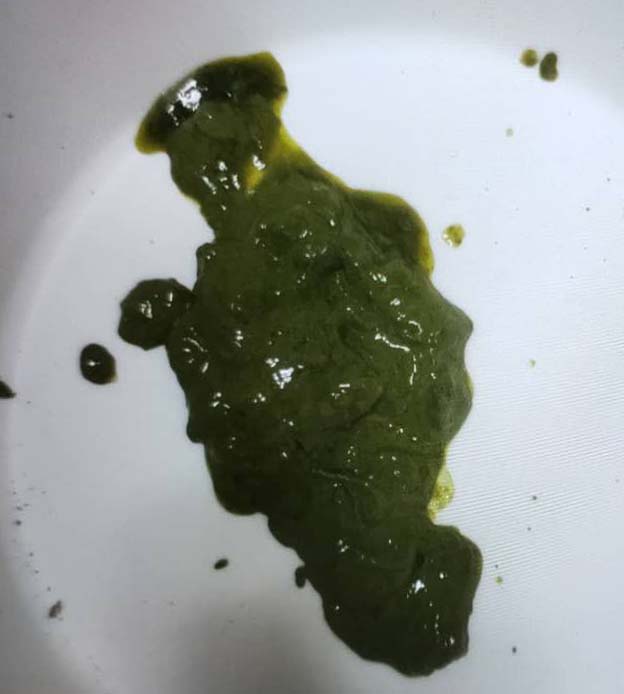

56. Which of the following stool colors is abnormal?（）
57.
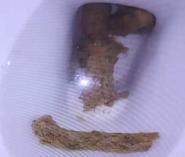

58.
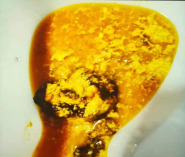

59.
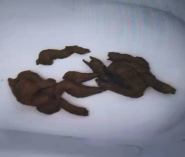

60.
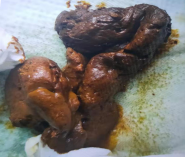

61. What is the most common type of constipation?（）
62. Functional constipation
63. Intestinal tuberculosis
64. Drug induced constipation
65. Crohn's disease
66. When constipation occurs, which of the following is wrong?（）
67. Increase the intake of vegetables, melons and fruits
68. Form the habit of defecating regularly
69. Improve mood
70. Reduce meat and fat intake
71. Where does the color of normal feces come from?（）
72. Food
73. Bilirubin produced by human body
74. Bile acids produced by the human body
75. Produced by intestinal bacteria
76. When diarrhea occurs, which of the following is wrong?（）
77. Light liquid food and less residue diet
78. Supplement water, mainly salty
79. Temperance
80. Increase the intake of vegetables, melons and fruits
81. What's wrong with green manure?（）
82. It's the normal stool color
83. It's a manifestation of disease
84. It is caused by the rapid passage of intestinal content through the intestine
85. If it persists, seek medical attention in time
86. When constipation, which laxative may cause melanosis coli?（）
87. Irritant laxative
88. Lactulose
89. Polyethylene glycol
90. Glycerin enema
91. Which drug is not recommended for routine use in diarrhea?（）
92. Loperamide
93. Montmorillonite powder
94. Bifidobacterium
95. Lactobacillus
96. Which of the following feces is abnormal?（）
97.
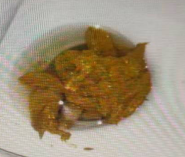

98.
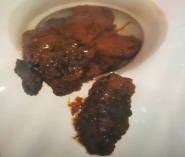

99.
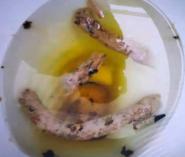

100.
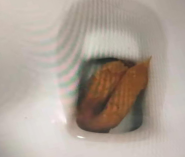

101. What are the reasons for the appearance of white feces or the lightening of feces?（）
102. Decreased bilirubin entering the intestine
103. Decreased bile acids entering the intestine
104. Reduction of intestinal bacteria
105. Eat a lot of white food
106. What is the disease that excretes white feces?（）
107. Bile duct stone
108. Inflammatory bowel disease
109. Cholecystitis
110. Gastric ulcer
111. Which of the following symptoms are associated with white feces or light feces?（）
112. Jaundice
113. Gastrointestinal bleeding
114. Fever
115. Constipation
116. When white feces appear, what is the purpose of MRCP?（）
117. Determine whether there is biliary obstruction
118. Determine if there is enteritis
119. Determine if there is cholecystitis
120. Determine if there is cirrhosis
121. When white feces appear, which of the following methods has the role of both examination and treatment?（）
122. ERCP
123. MRCP
124. CT
125. Ultrasound
126. The elderly people with bloody stool, accompanied by recent changes in defecation habits, must be alert to what diseases?（）
127. Colon cancer
128. Hemorrhoids
129. Ulcerative colitis
130. Colonic polyp
131. Bloody stool is accompanied by thinning of feces. What disease should we be vigilant against?（）
132. Colon cancer
133. Hemorrhoids
134. Ulcerative colitis
135. Colonic polyp
136. There is blood in the stool during defecation and blood drops after defecation. The blood is not mixed with feces. May it be?（）
137. Colon cancer
138. Hemorrhoids
139. Ulcerative colitis
140. Colonic polyp
141. In case of bloody stool, what tests should be improved?（）
142. Colonoscopy
143. Abdominal CT
144. Gastrointestinal radiography
145. Ultrasound
