# Supplementary material for: A preliminary study of further attempt at the development, testing and application of an independent primary screening stool card
Source: Sci Rep. 2022 Dec 21;12:22046. doi: 10.1038/s41598-022-26649-2 (PMC9768403; doi:10.1038/s41598-022-26649-2)
Supplement: Supplementary file 3 — Supplementary Information 3. [file 41598_2022_26649_MOESM3_ESM.docx]

**Clinical situation simulation test**

**1、Male, 65 years old, with a history of hepatitis B cirrhosis, excreted a large amount of black stool after eating（20 points）**

（1）What is the cause of melena? Where is the possible location of the disease?（4 points）

（2）What tests may be required for admission? Describe the purpose of each inspection?（10 points）

（3）The treatment principle of this disease?（6 points）

**2、Female, 45 years old, jaundice, with dark yellow urine and acholic stool.（20 points）**

（1）What are the reasons of acholic stool? and what are the possible causes?（4 points）

（2）What tests may be required for admission? Describe the purpose of each inspection?（10 points）

（3）The treatment principle of this disease?（6 points）

**3、Male, 20 years old, who recently ate unclean diet, defecated more than 3 times a day, and the excreted feces were watery stool.（10 points）**

（1）What disease is currently under consideration？（2 points）

（2）What measures can be taken after symptoms appear?（8 points）

**4、Male, 50 years old, with less than 3 defecation times per week in the past six months, accompanied by difficult defecation and dry and hard stool shape.（10 points）**

（1）What disease is currently under consideration？（2 points）

（2）What measures can be taken after symptoms appear?（8 points）

**5、Female, 30 years old, has blood when defecating, blood is not mixed with feces, attached to the surface of feces, and there is no obvious pain when defecating.（10 points）**

（1）What is the disease most likely to cause this symptom? What is the best way to diagnose the cause? (4 points)

（2）What are the means to treat this disease? (6 points)
