# Supplementary material for: A preliminary study of further attempt at the development, testing and application of an independent primary screening stool card
Source: Sci Rep. 2022 Dec 21;12:22046. doi: 10.1038/s41598-022-26649-2 (PMC9768403; doi:10.1038/s41598-022-26649-2)
Supplement: Supplementary file 4 — Supplementary Information 4. [file 41598_2022_26649_MOESM4_ESM.doc]

The Result of the Version for Patients	
	Attitude	Total	
	Strongly Agree	Agree	Neutral	Disagree	Strongly Disagree		
Question	Q1	Count	15	9	3	2	4	33	
		% within Question	45.5%	27.3%	9.1%	6.1%	12.1%	100.0%	
	Q2	Count	17	8	4	2	2	33	
		% within Question	51.5%	24.2%	12.1%	6.1%	6.1%	100.0%	
	Q3	Count	6	10	10	5	2	33	
		% within Question	18.2%	30.3%	30.3%	15.2%	6.1%	100.0%	
	Q4	Count	15	10	4	3	1	33	
		% within Question	45.5%	30.3%	12.1%	9.1%	3.0%	100.0%	
	Q5	Count	16	7	7	1	2	33	
		% within Question	48.5%	21.2%	21.2%	3.0%	6.1%	100.0%	
	Q6	Count	6	13	8	2	4	33	
		% within Question	18.2%	39.4%	24.2%	6.1%	12.1%	100.0%	
	Q7	Count	13	11	6	1	2	33	
		% within Question	39.4%	33.3%	18.2%	3.0%	6.1%	100.0%	
	Q8	Count	20	7	1	4	1	33	
		% within Question	60.6%	21.2%	3.0%	12.1%	3.0%	100.0%	
	Q9	Count	6	4	2	10	11	33	
		% within Question	18.2%	12.1%	6.1%	30.3%	33.3%	100.0%	
	Q10	Count	16	6	7	2	2	33	
		% within Question	48.5%	18.2%	21.2%	6.1%	6.1%	100.0%	
	Q11	Count	10	16	2	2	3	33	
		% within Question	30.3%	48.5%	6.1%	6.1%	9.1%	100.0%	
	Q12	Count	11	11	6	3	2	33	
		% within Question	33.3%	33.3%	18.2%	9.1%	6.1%	100.0%	
	Q13	Count	2	1	4	11	15	33	
		% within Question	6.1%	3.0%	12.1%	33.3%	45.5%	100.0%	
	Q14	Count	8	5	8	10	2	33	
		% within Question	24.2%	15.2%	24.2%	30.3%	6.1%	100.0%	
	Q15	Count	7	17	4	4	1	33	
		% within Question	21.2%	51.5%	12.1%	12.1%	3.0%	100.0%	
	Q16	Count	10	14	2	3	4	33	
		% within Question	30.3%	42.4%	6.1%	9.1%	12.1%	100.0%	
	Q17	Count	9	17	4	2	1	33	
		% within Question	27.3%	51.5%	12.1%	6.1%	3.0%	100.0%	
Total	Count	187	166	82	67	59	561	
	% within Question	33.3%	29.6%	14.6%	11.9%	10.5%	100.0%	


The Result of The Version for Health Service Provider	
	Attitude	Total	
	Strongly Agree	Agree	Neutral	Disagree	Strongly Disagree		
Question	Q1	Count	8	9	1	1	1	20	
		% within Question	40.0%	45.0%	5.0%	5.0%	5.0%	100.0%	
	Q2	Count	5	11	2	1	1	20	
		% within Question	25.0%	55.0%	10.0%	5.0%	5.0%	100.0%	
	Q3	Count	6	8	4	1	1	20	
		% within Question	30.0%	40.0%	20.0%	5.0%	5.0%	100.0%	
	Q4	Count	7	7	1	3	2	20	
		% within Question	35.0%	35.0%	5.0%	15.0%	10.0%	100.0%	
	Q5	Count	5	6	4	4	1	20	
		% within Question	25.0%	30.0%	20.0%	20.0%	5.0%	100.0%	
	Q6	Count	5	6	4	3	2	20	
		% within Question	25.0%	30.0%	20.0%	15.0%	10.0%	100.0%	
	Q7	Count	6	6	3	1	4	20	
		% within Question	30.0%	30.0%	15.0%	5.0%	20.0%	100.0%	
	Q8	Count	8	8	2	1	1	20	
		% within Question	40.0%	40.0%	10.0%	5.0%	5.0%	100.0%	
	Q9	Count	1	2	2	7	8	20	
		% within Question	5.0%	10.0%	10.0%	35.0%	40.0%	100.0%	
	Q10	Count	7	6	5	1	1	20	
		% within Question	35.0%	30.0%	25.0%	5.0%	5.0%	100.0%	
	Q11	Count	3	7	5	2	3	20	
		% within Question	15.0%	35.0%	25.0%	10.0%	15.0%	100.0%	
	Q12	Count	6	5	4	4	1	20	
		% within Question	30.0%	25.0%	20.0%	20.0%	5.0%	100.0%	
	Q13	Count	2	2	5	6	5	20	
		% within Question	10.0%	10.0%	25.0%	30.0%	25.0%	100.0%	
	Q14	Count	4	4	10	2	0	20	
		% within Question	20.0%	20.0%	50.0%	10.0%	0.0%	100.0%	
	Q15	Count	5	10	2	1	2	20	
		% within Question	25.0%	50.0%	10.0%	5.0%	10.0%	100.0%	
	Q16	Count	5	9	2	3	1	20	
		% within Question	25.0%	45.0%	10.0%	15.0%	5.0%	100.0%	
	Q17	Count	6	6	6	1	1	20	
		% within Question	30.0%	30.0%	30.0%	5.0%	5.0%	100.0%	
Total	Count	89	112	62	42	35	340	
	% within Question	26.2%	32.9%	18.2%	12.4%	10.3%	100.0%	
