# Supplementary material for: A preliminary study of further attempt at the development, testing and application of an independent primary screening stool card
Source: Sci Rep. 2022 Dec 21;12:22046. doi: 10.1038/s41598-022-26649-2 (PMC9768403; doi:10.1038/s41598-022-26649-2)
Supplement: Supplementary file 5 — Supplementary Information 5. [file 41598_2022_26649_MOESM5_ESM.doc]

Level * Attitude Crosstabulation( Freshman )	
	Attitude	Total	
	Overall Agree	Neutural	Overall Disagree		
Level	Grade A	Count	152	44	59	255	
		Expected Count	160.5	37.3	57.3	255.0	
	Grade B	Count	201	38	67	306	
		Expected Count	192.5	44.7	68.7	306.0	
Total	Count	353	82	126	561	
	Expected Count	353.0	82.0	126.0	561.0	
Chi-Square Tests	
	Value	df	Asymptotic Significance (2-sided)	
Pearson Chi-Square	3.138	2	.208	
Likelihood Ratio	3.129	2	.209	
Linear-by-Linear Association	1.078	1	.299	
N of Valid Cases	561			

Title * Attitude Crosstabulation( Clinicians )	
	Attitude	Total	
	Overall Agree	Neutural	Overall Disagree		
Title	Attending Physician	Count	115	44	45	204	
		Expected Count	120.6	37.2	46.2	204.0	
	Deputy Chief Physician	Count	40	11	17	68	
		Expected Count	40.2	12.4	15.4	68.0	
	Chief Physician	Count	46	7	15	68	
		Expected Count	40.2	12.4	15.4	68.0	
Total	Count	201	62	77	340	
	Expected Count	201.0	62.0	77.0	340.0	
Chi-Square Tests	
	Value	df	Asymptotic Significance (2-sided)	
Pearson Chi-Square	5.058	4	.281	
Likelihood Ratio	5.400	4	.249	
Linear-by-Linear Association	.752	1	.386	
N of Valid Cases	340			
